# Electrical impedance tomography to set positive end-expiratory pressure

**DOI:** 10.1097/MCC.0000000000001255

**Published:** 2025-02-12

**Authors:** Juliette E. Francovich, Bhushan H. Katira, Annemijn H. Jonkman

**Affiliations:** a Department of Adult Intensive Care, Erasmus Medical Center, Rotterdam, The Netherlands; b Department of Pediatrics, Washington University in St. Louis, School of Medicine, St. Louis, Missouri, USA

**Keywords:** electrical impedance tomography, lung mechanics, positive end-expiratory pressure, right ventricle function

## Abstract

**Purpose of review:**

To summarize the rationale and concepts for positive end-expiratory pressure (PEEP) setting with electrical impedance tomography (EIT) and the effects of EIT-based PEEP setting on cardiopulmonary function.

**Recent findings:**

EIT allows patient-specific and regional assessment of PEEP effects on recruitability and overdistension, including its impact on ventilation-perfusion (V̇/Q) mismatch. The overdistension and collapse (OD-CL) method is the most used EIT-based approach for PEEP setting. In the RECRUIT study of 108 COVID-19 ARDS patients, the PEEP level corresponding to the OD-CL crossing point showed low overdistension and collapse (below 10% and 5%, respectively) regardless of recruitability. In a porcine model of acute respiratory distress syndrome (ARDS), it was shown that at this crossing point, respiratory mechanics (compliance, Δ*P*) were consistent, with adequate preload, lower right ventricular afterload, normal cardiac output, and sufficient gas exchange. A recent meta-analysis found that EIT based PEEP setting improved lung mechanics and potentially outcomes in ARDS patients. EIT thus provides critical insights beyond respiratory mechanics and oxygenation for individualized PEEP optimization. EIT-based methods for PEEP setting during assisted ventilation have also been proposed.

**Summary:**

EIT is a valuable technique to guide individualized PEEP setting utilizing cardiopulmonary information that is not captured by respiratory mechanics and oxygenation response alone.

## INTRODUCTION

Lung-protective ventilation strategies involve adequate positive end-expiratory pressure (PEEP) setting while maintaining effective gas exchange [1–3]. With recruitable lungs, high PEEP can maximize lung aeration and prevent the repetitive opening/closing of alveoli and small airways, thereby minimizing atelectrauma and improving oxygenation [2–5]. However, in nonrecruitable or poorly recruitable lungs, excessive PEEP may cause lung overdistension and further injury [2] as well as cardiovascular impairment [6]. This could worsen ventilation-perfusion (V̇/Q) mismatch, a factor associated with higher risk of ventilator-induced lung injury (VILI) and increased mortality [7,8].

A major challenge in PEEP management is the variable individual patient's response to higher pressures [4]. Beyond PEEP, other recruitment strategies such as prone positioning [9] also vary in its effects on V̇/Q matching, lung mechanics, and gas exchange [10]. Consequently, there is no universally superior PEEP-setting strategy [11^▪^,^12^^▪▪^,^13^], warranting an individualized approach and periodic re-evaluation including with positioning changes [10]. Methods based on oxygenation, respiratory mechanics, or esophageal pressure have not consistently proven superior to improve outcomes [11^▪^,^12^^▪▪^,^13^]. The inconclusive results are likely partly explained by the lack of a reliable method to assess recruitability and overdistension at the bedside [14].

The recruitment-to-inflation (R/I) ratio is a relatively new bedside tool for identifying potential PEEP responders [15,16]. However, it does not suggest the “optimal” PEEP level and typically relies on a single derecruitment step (e.g., reducing PEEP from 15 to 5 cmH_2_O), limiting its granularity for PEEP setting [16,17]. Additionally, it does not reflect regional recruitment or overdistension [16,18]. Electrical impedance tomography (EIT) can noninvasively estimate both recruitability and overdistension [11^▪^,^12^^▪▪^]. It is an attractive bedside tool for PEEP setting as it allows assessing the patient-specific and regional (e.g., dependent vs. nondependent lung) response to PEEP and the interaction with position changes [10,11^▪^,^12^^▪▪^], as well as the effects of PEEP on V̇/Q mismatch [7,19]. Moreover, in a recruitable model of acute respiratory distress syndrome (ARDS), the total respiratory system compliance behaved like the dorsal lung compliance and the PEEP set to achieve best compliance was associated with overdistension of the ventral lung [20], suggesting a need for bedside assessment of collapse and overdistension while setting PEEP.

This review describes different approaches for EIT-based PEEP setting and its relation to other physiological parameters and clinical outcomes, focusing on new developments. Considering the importance of heart-lung interactions, we also detail the role of EIT for evaluating and enhancing both respiratory and cardiovascular function during PEEP selection. 

**Box 1:**
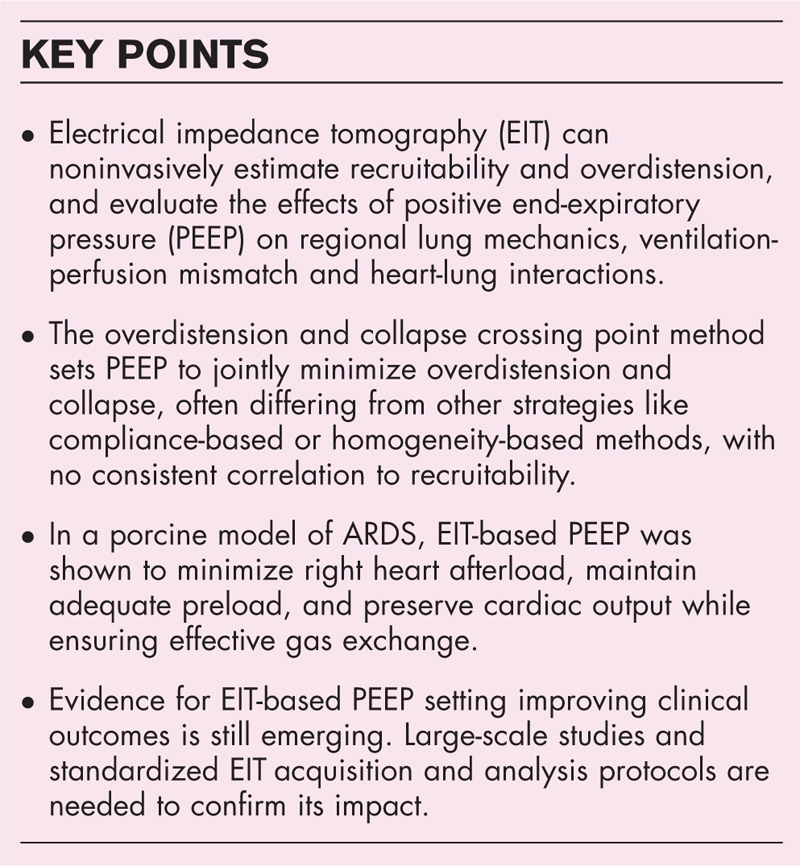
no caption available

## DIFFERENT APPROACHES TO POSITIVE END-EXPIRATORY PRESSURE SETTING WITH ELECTRICAL IMPEDANCE TOMOGRAPHY

For background on the EIT technique and clinical applications, we refer to earlier reviews [21,22^▪▪^,^23^]. EIT is an attractive modality for individualized PEEP selection since changes in end-expiratory lung volume (EELV) are closely correlated with changes in end-expiratory lung impedance (EELI) [24–26]: an increase in PEEP will result in an increase in EELI. Assessing changes in EELI can therefore track changes in EELV following recruitment maneuvers [27], without requiring a full PEEP trial. It should be noted, however, that EELI is measured in arbitrary units, depending on device calibrations, and may also change because of artifacts, patient movements and changes in fluid status [22^▪▪^]. Therefore, absolute EELI and ΔEELI cannot be utilized and compared between recordings/patients to estimate absolute recruited lung volumes.

Various computational methods have been reported utilizing EIT information for personalized PEEP selection, detailed in Table 1.

**Table 1:** Overview of different methods for PEEP setting with EIT, their physiological rationale and considerations for clinical use. The methods are divided into methods that can only be used or have only been validated during controlled mechanical ventilation and methods that can be used during assisted mechanical ventilation with spontaneous patient effort

Method	Description	Physiological rationale	Considerations
Controlled mechanical ventilation			
OD-CL method	Assesses changes in pixel compliance during a decremental PEEP trial to determine the effects of PEEP on regional collapse and overdistension [36]	Jointly minimizing collapse and overdistension based on pixel-level compliance [36]	Available on most commercial devices and most studied approach [74^▪^]
Silent space analysis	Identifies silent spaces (regions with amplitude <10% of maximal pixel amplitude) in a decremental PEEP trial to locate collapsed or overdistended lung areas [39–41]	Silent spaces above the CoV are nondependent silent spaces and indicate overdistension, whereas silent spaces below the CoV are dependent silent spaces and indicate collapse [39]	Only available on devices that apply lung contouring and results with generic lung contours should be interpreted with caution [78]
GI method	Calculates the GI [42] to assess lung inhomogeneity at each PEEP level during a PEEP trial [43]	Minimizing regional inhomogeneity and achieving better global lung ventilation [42,43]	Described using extended incremental PEEP trials (e.g., 30 min per PEEP step) [43]; time-consuming and labor-intensive
Regional compliance	Measures compliance during sustained inflation maneuver and PEEP increase to assess recruitability in four horizontal ROIs [38]. Measures compliance during driving pressure (Δ*P*) decrease to assess alveolar cycling and overdistension [38]	Allows for region-specific adjustments in PEEP. Compliance increase in any ROI after PEEP increase indicates recruitment [38]. Compliance increase in any ROI after ΔP decrease indicates overdistention at original ΔP [38]	Requires sustained inflation maneuvers [38]; Requires ROI selection, which is not an arbitrary task [47]
EELI method	Measures changes in end-expiratory lung impedance (EELI) following recruitment maneuvers, guiding PEEP adjustments based on EELI changes over time [27]	Tracks recruitment stability, adjusting PEEP to maintain lung recruitment [27]	Involves repeated recruitment maneuvers, which may not be recommended for ARDS patients [3,79,80]; EELI is sensitive to nonventilation related artefacts [81]
CoV approach	Ensures even distribution of ventilation between dependent and nondependent lung regions by finding the PEEP level in which the CoV is closest to 50% [44]	Too low PEEP resulting in collapse in the dependent lung results in predominantly nondependent ventilation (CoV < 50%) [44]. Too high PEEP resulting in overinflation of the nondependent lung results in a shift in ventilation to the dependent lung (CoV > 50%) [44]	Cannot account for differences between the left and right lung in asymmetrical lung injury; Setting PEEP to achieve CoV near 50% may not be optimal, as it risks overdistension in ventral lung regions [59]. Instead, a lower, more ventral CoV position could be more suitable but should be studied further [59]
Ventral-to-dorsal ratio	Classifies ventro-dorsal ventilation distribution phenotypes to assess the optimal PEEP to prevent overdistension and collapse [45]	Using PEEP to achieve a more homogenous ventro-dorsal ventilation distribution reduces risk of pulmonary complications in postoperative patients [45]	Might be less reliable in cases where focal lung disease and PEEP settings both impact ventilation distribution [46]; Requires ROI selection, which is not an arbitrary task [47]
Assisted mechanical ventilation			
Regional peak flow (RPF)	The maximum first derivative (i.e., regional peak flow) per pixel is calculated per PEEP level. Based on the RPF, the cumulative overdistension and collapse rates are then calculated similar to the original OD-CL algorithm [49,50]	This method assumes that RPF explains regional pulmonary compliance and can therefore be used to minimize overdistension and collapse rates [49]	This method can be applied in spontaneously breathing patients since RPF does not rely on a zero-flow state to achieve equally distributed airway pressure [49]
EIT integrated with transpulmonary pressure	Assesses changes in dynamic pixel compliance (pixel TIV/dynamic transpulmonary driving pressure) during a decremental PEEP trial [54]. The cumulative overdistension and collapse rates are then calculated similar as in the original OD-CL algorithm [54]	By integrating dynamic transpulmonary driving pressure, this algorithm accounts for patient effort [54]	This method can be applied in spontaneously breathing patients since the transpulmonary pressure allows to assess respiratory mechanics with active patient effort [54,82]

Δ*P*, driving pressure; CoV, center of ventilation; EELI, end-expiratory lung impedance; EIT, electrical impedance tomography; GI, global inhomogeneity; OD-CL, overdistension and lung collapse; PEEP, positive end-expiratory pressure; ROI, region of interest; RPF, regional peak flow; TIV, tidal impedance variation.

### Overdistension and collapsed lung method

**FIGURE 1:**
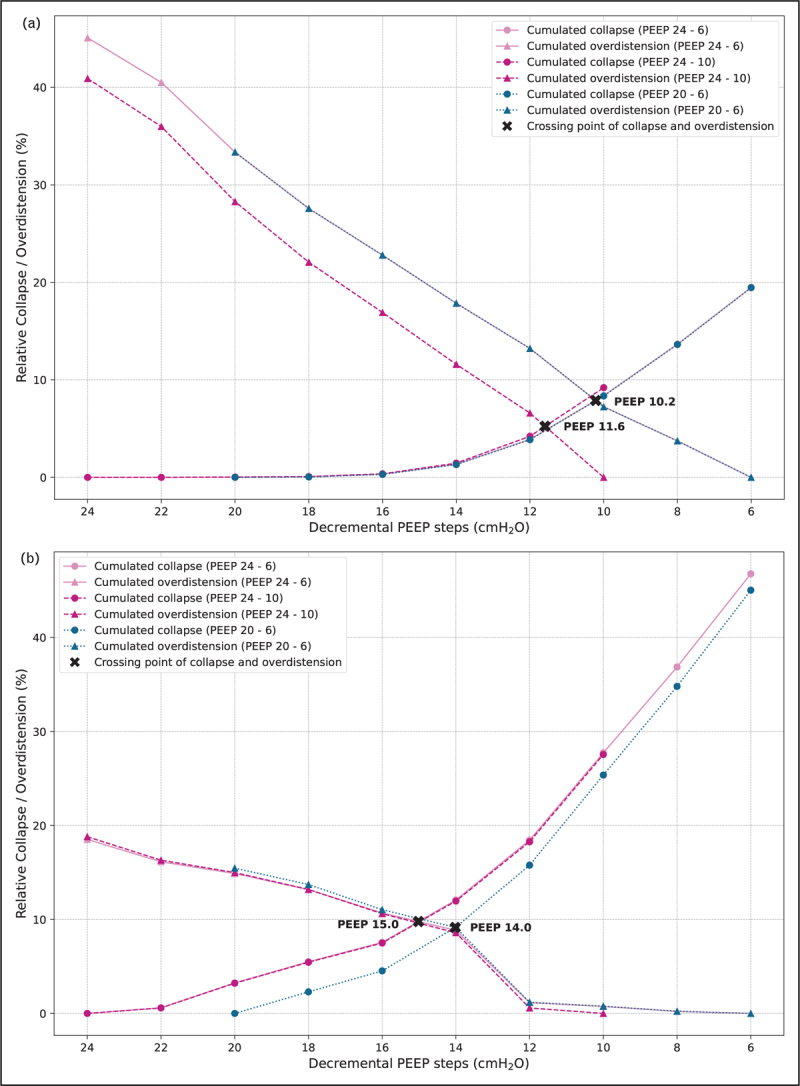
This figure gives two illustrative examples of the effect of the selected PEEP range during a decremental PEEP trial on the crossing point between the cumulated collapse and overdistension curves, as determined by the Costa approach. In both patients, the full PEEP trial was performed from 24 to 6 cmH_2_O. (a) In patient A, shortening the PEEP trial at the lower PEEP levels (24–10 cmH_2_O) resulted in a 1.4 cmH_2_O increase in the PEEP level at the crossing point, while shortening the PEEP trial at the higher PEEP levels (20–6 cmH_2_O) had no effect on the crossing point, since the relative collapse is very stable in the range from 24 to 20 cmH_2_O and thus does not influence the crossing point. (b) Conversely, in patient B, shortening the PEEP trial at the lower PEEP levels did not result in a shift in the crossing point, while shortening the PEEP trial at the higher PEEP levels resulted in a 1 cmH_2_O decrease in the PEEP level at the crossing point.

The most commonly used method, especially in patients with ARDS [11^▪^,^28^–^33^] including those on veno-venous ECMO [34], but now also gaining ground for intraoperative PEEP setting [35], is the overdistension and collapse (OD-CL) method, first introduced by Costa *et al.*[36]. This method assesses relative changes in regional compliance on a pixel-level during a decremental PEEP trial, requiring the measurement of driving pressure (Δ*P*) [36]. It assumes that every pixel has its optimal compliance [measured as tidal impedance variation (TIV) divided by Δ*P*] at some pressure within the applied PEEP range. Relative compliance loss towards higher PEEP is interpreted as overdistension, whereas relative compliance loss at lower PEEP is interpreted as collapse [36] – with the highest and lowest applied PEEP serving as reference for 0% collapse and 0% overdistension, respectively. Optimal PEEP is then set at the crossing point of the overdistension and collapse curves, aimed at jointly minimizing both phenomena [36]. Since relative changes in compliance are computed, the crossing point depends on the applied PEEP range (Fig. 1), and therefore a wide range is recommended for standardization [22^▪▪^] – e.g. from 24 to 6 cmH_2_O, when clinically acceptable. For further recommendations for a standardized EIT-guided PEEP trial with OD-CL method, we refer to [22^▪▪^]. Moreover, position changes, such as from supine to prone position, could also influence the amount of collapse and overdistension because of PEEP. For instance, prone position resulted in lower collapse at low PEEP levels but higher overdistension in PEEP above 10 cmH_2_O [37]. Repeating PEEP trials after a clinical change or a position change should be considered.

### Other approaches to positive end-expiratory pressure setting in controlled ventilation

Various alternative approaches for assessing lung mechanics at different PEEP levels exist (Table 1). For instance, PEEP has been adjusted by measuring changes in compliance across four horizontal lung regions after a PEEP increase or Δ*P* decrease [38]. The silent spaces method identifies hypoventilated regions within a lung contour [39–41], based on a cut-off in tidal impedance changes. One potential advantage of this approach is that it does not require measurement of regional compliance, which typically necessitates ventilator-controlled breathing and knowledge of Δ*P*. The Global Inhomogeneity (GI) index has been utilized to minimize inhomogeneity and optimize overall ventilation [42,43] and the Center of Ventilation (CoV) approach ensures balanced ventilation between dependent and nondependent lung regions [44]. Similarly, the ventral-to-dorsal ratio has been used to classify the ventilation distribution to assess the optimal PEEP level [45]. While EIT phenotyping of ventilation distribution patterns can be beneficial in postoperative patients with relatively healthy lungs, it may be less reliable in cases where focal lung disease and PEEP settings both impact ventilation distribution [46]. Notably, CoV has been proposed as a more robust and sensitive parameter for assessing ventro-dorsal ventilation distribution [23], as it avoids the need for region of interest selection, which can significantly influence ventral-to-dorsal ratio results [47].

### Electrical impedance tomography-based positive end-expiratory pressure setting during spontaneous breathing

EIT application in spontaneously breathing patients present additional challenges due to variable breathing patterns and increased movement artifacts [48]. Many of the above parameters require a uniform airway pressure phase or known Δ*P* and have not been validated for spontaneous breathing [49,50]. In spontaneous breathing, higher PEEP levels might decrease the patient's effort depending on whether PEEP enhances lung compliance or results in overdistension [51,52]. Few EIT parameters have been explored for PEEP setting during spontaneous breathing (Table 1). For example, the Regional Peak Flow (RPF) method was recently introduced [49,50] and estimates RPF as the maximum first derivative of the inspiratory limb of the impedance signal, assuming RPF reflects regional compliance [49]. Relative changes in RPF are then used to estimate overdistension and collapse, similar to the OD-CL approach [49]. However, the accuracy of this method depends heavily on the filtering used to remove artifacts, as different filters affect the amplitude and timing of the EIT signal [53]. An alternative approach is to integrate EIT with dynamic transpulmonary driving pressure (Δ*P*_L_) to calculate dynamic lung compliance per pixel, accounting for the impact of patient effort on lung mechanics [54]. In a randomized crossover trial [55^▪^], PEEP set based on EIT and Δ*P*_L_ (PEEP_EIT_Δ*P*L_) was compared to PEEP set according to the low PEEP/FiO_2_ table in patients on pressure support ventilation. PEEP_EIT_Δ*P*L_ resulted in lower dynamic lung stress and work of breathing due to a reduction in respiratory drive and less alveolar collapse with limited increase in overdistension [55^▪^]. One drawback is that this method requires both EIT and synchronized esophageal manometry [55^▪^], and the relevance of complex PEEP titration in patients with mild ARDS could be questioned. Alternative simple bedside methods like occlusion pressure-based PEEP selection aligned better with EIT than the PEEP/FiO_2_ table, but there was significant patient-level variability [55^▪^].

## OD-CL CROSSING POINT METHOD VS. OTHER POSITIVE END-EXPIRATORY PRESSURE SETTING STRATEGIES

In a large observational study in 108 COVID-19 ARDS patients (RECRUIT study) [11^▪^], it was found that at the PEEP level corresponding to the OD-CL crossing point, respiratory mechanics (compliance, Δ*P*) were similar among patients and overdistension and collapse were low (below 10% and 5%, respectively), regardless of the amount of recruitability [11^▪^]. The PEEP level at the crossing point was found to correspond to a slightly positive end-expiratory transpulmonary pressure (P_L_) [56], supporting the concept of maintaining open lung units without excessive pressures [11^▪^,^57^]. Interestingly, PEEP setting based on P_L_ differed significantly from PEEP setting with EIT when minimizing silent spaces rather than using the OD-CL crossing point [40,41]. In comparison, in 15 ARDS patients, methods based on the highest respiratory system compliance tended to select slightly lower PEEP levels than the OD-CL crossing point, while strategies based on the CoV and maximum plateau pressure of 28–30 cmH_2_O (i.e., *Express* strategy) resulted in higher PEEP [12^▪▪^].

Discrepancy between the PEEP corresponding to the OD-CL crossing point vs. optimal respiratory system compliance was also noted in the RECRUIT cohort [11^▪^], where in only 20/108 patients both methods suggested the same optimal PEEP. Moreover, GI-based PEEP setting often resulted in higher PEEP levels than the OD-CL approach [58], likely promoting overdistension when achieving homogenization.

Optimal PEEP levels resulting from different methods were not associated with recruitability as determined by the R/I-ratio [12^▪▪^]. However, this should be interpreted with caution as the R/I-ratio fails to capture information on overdistension which might explain the lack of correlation with EIT-based optimal PEEP [16,18,59]. Changes in EELV as captured by EIT can be used to calculate a regional R/I-ratio, leveraging the combination of this simple bedside maneuver with regional information [60,61].

## NEW INSIGHTS FROM PRE-CLINICAL STUDIES

The OD-CL approach aims to jointly minimize both overdistension and collapse, assuming that both are equally harmful in terms of contributing to VILI. However, recent experimental data suggest that this may not be true in some conditions [62^▪▪^,^63^,^64^], with collapse/atelectasis potentially having more detrimental consequences to the lungs and right heart, and resulting in worsening of alveolar-capillary barrier function especially in the presence of systemic inflammation [64]. In a porcine model of ARDS, EIT-based PEEP strategies that allowed various levels of collapse were tested: OD-CL crossing point method, ≤3% collapse, or ≤3% overdistension [62^▪▪^]. Low tidal volume protective ventilation was applied for 12 h. Notably, in the minimal overdistension group, 6/12 pigs died prior to experiment completion, likely because of cardiovascular compromise and right heart failure. Allowing minimal overdistension inherently means accepting higher levels of collapse, which was present for approximately 25% of lung units and associated with poor respiratory mechanics, and higher intrapulmonary shunt and lung injury on histology [62^▪▪^]. This suggests that setting PEEP on the crossing point or on the least amount of collapse is preferable.

The OD-CL method was also compared with blood gas-based PEEP titration in a porcine lung injury model [65], evaluating whether dead space (Vd/Vt) and intrapulmonary shunt (Qs/Qt) were associated with respectively overdistension and collapse on EIT during a decremental PEEP trial. With lowering PEEP, collapse and Qs/Qt increased in a similar matter, whereas changes in Vd/Vt did not reflect overdistension [65]. However, dead space was measured with the Enghoff equation instead of volumetric capnography and is therefore confounded by shunting effects. Nevertheless, this is an interesting approach that integrates effects on macro-circulation and micro-circulation during EIT-guided PEEP setting. The variety in responses observed highlight the need for a personalized approach.

## ELECTRICAL IMPEDANCE TOMOGRAPHY-GUIDED POSITIVE END-EXPIRATORY PRESSURE SETTING AND CARDIAC FUNCTIONING

EIT-based PEEP setting should not only consider the effects of PEEP on regional lung mechanics, but also on hemodynamics. From a physiological perspective, application of positive airway pressure increases pleuro-pericardial pressures as well as P_L_ (i.e., airway – pleural pressure) [66–68]. Increase in juxta-cardiac pleural pressure raises the pericardial pressure and compresses the right heart, reducing its ability to relax with in-flow of blood (i.e., lowering preload) [66,67]. On the other hand, increases in P_L_ increases the West zone I-II conditions of the lung which increases the right heart afterload [68]. The combination of lower preload and higher afterload of the right heart results in reduced left heart preload and stroke volume [69,70]. Mechanical factors that dictate this physiological behavior include the level of PEEP and Δ*P* set on the ventilator, and the degree of lung overdistension or collapse. Overdistended lungs (either because of high PEEP, or Δ*P* or poor potential for recruitment) are likely to increase the juxta-cardiac pleural pressure reducing the preload on one hand, and given the increase in lung zones I and II, also increases the afterload. In contrast, collapsed lung will allow for more preload and lower afterload due to mechanical load, but may cause intra-alveolar hypoxemia and arterial vasoconstriction leading to high afterload and possibly rapid decline in right heart function [70,71]. This suggests that the mechanical advantage for the lungs is necessary for the mechanical advantage of heart. EIT provides an excellent opportunity to monitor both effects. Indeed, the crossing point PEEP was accompanied with lower right ventricular afterload, adequate preload and normal cardiac output, as well as sufficient gas exchange [62^▪▪^].

### Assessing pulsatility and perfusion with electrical impedance tomography

Thoracic impedance changes primarily result from changes in lung volume, but also include a cardiovascular pulsatile component that can be extracted via filtering of the EIT signal [72]. Blood flow through the heart increases the impedance during systole and decreases during diastole; while reverse phase change happens when the blood flows through the pulmonary blood vessels. These changes are likely influenced by vessel tone and stroke volume. Increase in PEEP has been demonstrated to reduce the stroke volume measured via EIT [73]. On the other hand, as the lung opens with PEEP, local oxygenation and vasoconstriction improves, potentially improving pulsatile blood.

Perfusion is the distribution of blood in the pulmonary field and can be evaluated with EIT during a conductivity-contrasting bolus injection [22^▪▪^]. Together with the ventilation distribution image, this enables a bedside rapid and repeated ability to assess V̇/Q matching. Better V̇/Q matching with higher PEEP was observed in a small cohort of ARDS patients [7]. Studies addressing if the OD-CL crossing point PEEP will relate to the best V̇/Q match are underway.

## TOWARDS IMPROVING CLINICAL OUTCOMES

Despite the increased use and popularity of EIT, evidence that EIT-based PEEP setting improves clinical outcomes is still in an early stage. A recent meta-analysis found that the OD-CL approach improved respiratory mechanics (compliance) and potentially outcomes in patients with ARDS [74^▪^]. More recently, a single center phase-II randomized clinical trial in COVID-19 ARDS patients compared an EIT-guided strategy, aimed at minimizing Δ*P*, with PEEP setting using the ARDSnet low-PEEP/FiO_2_ table [75^▪^]. Although no differences in survival and ventilator weaning duration were found, the EIT-guided strategy suggested a faster improvement of lung function. In addition, this study showed the feasibility of an EIT-guided PEEP setting [75^▪^]. A large RCT in 376 patients aimed at improving 28-day mortality with EIT-guided PEEP setting is currently underway [76].

Key to developing and studying EIT-guided ventilation strategies is a standardized approach to implementing EIT in clinical practice, including protocols for patient selection, acquisition and interpretation of the data [77]. This will help to promote generalizability of research and we recently developed recommendations for a structured EIT application, including during a PEEP trial [22^▪▪^].

## CONCLUSION

EIT can greatly enhance our understanding of the regional distribution of ventilation and perfusion during PEEP titration. These physiological insights could guide ventilation management and various methods for EIT-based PEEP setting have been reported, with the OD-CL method most applied. Standardization is needed for developing and testing EIT-guided PEEP setting strategies that could potentially improve clinical outcomes – not only focusing on the lungs, but also on the heart and V̇/Q matching. EIT then has the potential to further evolve as an important technique for optimizing cardiopulmonary interaction while setting PEEP.

## Acknowledgements

*The authors thank P. Somhorst for providing the data from decremental PEEP trials in two COVID-19 ARDS patients forFig. 1*.

### Financial support and sponsorship

*No funding was received for this work. AJ is supported by a personal grant from NWO (ZonMw Veni 2022, 09150162210061, paid to the institution) and has received research funding (paid to the institution) from Pulmotech B.V. (Netherlands), Liberate Medical (Crestwood, Kentucky), Netherlands eScience Center (Netherlands) and Health∼Holland (Netherlands).*

### Conflicts of interest

*There are no conflicts of interest.*

## REFERENCES AND RECOMMENDED READING

Papers of particular interest, published within the annual period of review, have been highlighted as:

▪ of special interest
▪▪ of outstanding interest
